# A Survey of Living Conditions and Psychological Distress in Japanese University Freshmen during the COVID-19 Pandemic

**DOI:** 10.3390/healthcare11010094

**Published:** 2022-12-28

**Authors:** Yoko Iio, Yukihiro Mori, Yuka Aoyama, Hana Kozai, Mamoru Tanaka, Makoto Aoike, Hatsumi Kawamura, Manato Seguchi, Masato Tsurudome, Morihiro Ito

**Affiliations:** 1 Graduate School of Life and Health Sciences, Chubu University, 1200 Matsumoto-cho, Kasugai 487-8501, Japan; 2 Department of Lifelong Sports and Health Sciences, College of Life and Health Sciences, Chubu University, 1200 Matsumoto-cho, Kasugai 487-8501, Japan; 3 Center for Nursing Practicum Support, Chubu University, 1200 Matsumoto-cho, Kasugai 487-8501, Japan; 4 Department of Clinical Engineering, College of Life and Health Sciences, Chubu University, 1200 Matsumoto-cho, Kasugai 487-8501, Japan; 5 Department of Food and Nutritional Sciences, College of Bioscience and Biotechnology, Chubu University, 1200 Matsumoto-cho, Kasugai 487-8501, Japan; 6 Department of Biomedical Sciences, College of Life and Health Science, Chubu University, 1200 Matsumoto-cho, Kasugai 487-8501, Japan

**Keywords:** K6, psychological distress, living conditions, COVID-19 pandemic, university freshmen

## Abstract

Since the novel coronavirus disease 2019 (COVID-19) pandemic, educational institutions have implemented measures such as school closures, raising concerns regarding the increase in psychological distress among university students. The purpose of this study is to identify factors that have influenced psychological distress among college freshmen during the COVID-19 pandemic. A questionnaire survey was conducted at the conclusion of the sixth wave of COVID-19 in Japan. Psychological distress was measured using the six-item Kessler Psychological Distress Scale (K6). Factors affecting psychological distress were calculated using regression analysis. Of the 2536 participants, 1841 (72.6%) reported having no psychological distress, while 695 (27.4%) reported having psychological distress. Factors that were identified to contribute to psychological distress were lack of sleep, weight gain or loss, worsening of interpersonal relationships, and physical symptoms and illnesses. A willingness to join an athletic club and having an environment in which it is easy to discuss worries and anxieties with others were factors that were identified to hinder psychological distress. It is necessary for universities to offer enhanced supports for physical and interpersonal activities. Additionally, it is imperative to encourage students to look after their physical health and to actively utilize university-based consultation systems.

## 1. Introduction

In 2019, a novel coronavirus disease (COVID-19) was discovered in Wuhan, China. The infection spread globally and reached the proportions of a pandemic [1], forcing social life to undergo major changes. The highly contagious nature of the severe acute respiratory syndrome coronavirus 2 (SARS-CoV-2) necessitated immediate quarantine and lockdowns, among other measures. Educational institutions adopted several infection control measures by ensuring physical distancing, modifying school activities, utilizing COVID-19 testing and isolation, screening for symptoms, and movement monitoring [2]. One of these measures was school closures, with more than 160 countries implementing national school closures, affecting more than 87% of the global student population, according to UNESCO monitoring. Several other countries also enforced localized school closures [3]. Online lectures associated with school closures and the subsequent loss of opportunities for personal interaction with teachers and classmates led to severe isolation, anxiety, and depression [4]. These changes in educational institutions during the COVID-19 pandemic have adversely impacted not only the quality of education but also the psychological well-being of students and faculty members [3,5,6,7]. In addition, it has been pointed out that online lectures themselves worsened students’ mental health [8] and led to a negative attitude toward academic work [9]. Prior studies have found an increase in psychological burden in people who have experienced natural and human-made disasters [10]. After the terrorist attacks in the United States on 11 September 2001, significant psychological distress and depression was reported among residents near the World Trade Center [11,12]. Earlier pandemics, including severe acute respiratory syndrome (SARS) and Middle East respiratory syndrome coronavirus (MERS), have also been shown to affect mental health and quality of life [12,13].

To adopt effective measures against psychological distress caused by such disasters and pandemics, it is necessary to identify the factors that contribute to such distress. A survey of Chinese university students indicated that the level (extent) of spread of the COVID-19 infection worsens sleep quality and indirectly affects mental health, and that daily physical activity and adequate sleep are necessary to improve living conditions [14]. Other studies have reported that sleep, diet, physical activity, loneliness and the deterioration of relationships, alcohol consumption, and smoking have affected psychological distress during the COVID-19 pandemic [15,16,17,18,19]. However, there are no studies on psychological distress among Japanese university freshmen during the COVID-19 pandemic.

This study focuses on incoming university students in Japan in 2022. The target students experienced the COVID-19 pandemic in their first year of high school. Thus, they spent most of their high school life—the period of determining their career path, i.e., whether or not to pursue post-secondary education—and entered higher education during the pandemic. In addition, university freshman students have no experience of university life and are not affected by it. We thought that by investigating this group, the university’s response and countermeasures would be clarified. The purpose of this study is to investigate the extent of psychological distress in college freshmen and to identify factors associated with it, such as physical activity, sleep, and relationships with others. Based on the findings of this study, we believe that it will be possible to construct specific considerations and advice for students that will lead to a healthy university life during a pandemic.

## 2. Materials and Methods

### 2.1. Study Design and Data Collection

A cross-sectional study was conducted in this study. In mid-April 2022, a questionnaire was sent to 2789 new students of all faculties belonging to University A. A total of 2552 (91.5%) students responded, of which 2536 (99.5%) were valid responses. During this time, the sixth wave of COVID-19 in Japan was beginning to subside and the average number of newly infected persons per day was less than 50,000 nationwide. In addition, in the municipality of the university where the survey was conducted, the Japanese government’s movement restrictions to prevent the spread of the disease, which included activity restrictions, were lifted on 21 March 2022, in conjunction with the nationwide withdrawal of pandemic-related activity restrictions and safety measures.

Statistical power analysis using the software IBM SPSS version 28 (Statistical Package for Social Science, Chicago, IL, USA) was performed to confirm the sample size required for this study. A sample size of 147 was required for a power of 0.8, a significance level of 0.05, and 17 explanatory variables. The sample size of this study satisfies this requirement.

The study was conducted with the approval of the Ethical Review Committee of Chubu University (Approval No.: 20220001).

### 2.2. Survey Items

Data collection was performed using a web-based questionnaire designed using a blank Google form. There was a maximum of 30 questionnaire items in total. At the freshman orientation of the university, paper application guidelines were distributed and subjects were recruited. Students who agreed to participate accessed the web survey by reading the QR code of the URL of the Google Form with their smartphones. Information regarding basic attributes, such as gender, university department, and club activities, was collected (Table 1). Participants were also asked about their lifestyle (breakfast intake and sleep), physical health (any physical symptoms and/or illnesses currently under treatment), and living environment during the COVID-19 pandemic (e.g., interactions with people and environments, etc.). In this study, these items such as “lifestyle”, “physical health”, and “living environments” are defined as “living conditions.”

We assessed psychological distress using the six-item Kessler Psychological Distress Scale (K6), which uses a six-item scale to measure psychological distress (depression and anxiety) occurring in the past 30 days. The questions determine how often within the 30-day timeframe the respondent felt: (a) nervous, (b) hopeless, (c) restless or fidgety, (d) so depressed that nothing could cheer them up, (e) that any task would be an effort to perform, and (f) worthless. In this study, any sort of mental stress, depression, and anxiety is defined as psychological distress.

The K6 is scored on a 5-point scale (0 to 4 points) for each question. The total score ranges from 0 to 24 points, with higher scores indicating a higher likelihood of mood and anxiety disorders. Scores were classified as 0–4 points (no stress), 5–8 points (psychological stress-response equivalent), 9–12 points (mood and/or anxiety-disorder equivalent), and 13–24 points (severe mental disorder). Several previous studies using K6 have set a cut-off value of 5 points for K6 scores [4,17,20]. Therefore, in this study, the participants were classified into two groups according to their total K6 score, with a cut-off value of 5 points: one group “without” psychological distress, and one group “with” psychological distress [21]. To examine the reliability of the K6, Cronbach’s alpha value was calculated (Cronbach’s alpha  = 0.91).

The International Physical Activity Questionnaire-Short Version (IPAQ-SV) was used to assess daily physical activity. The IPAQ-SV consists of up to 7 items, and includes questions about the hours per day and days per week spent walking and doing high- or moderate-intensity physical activity in an average week. It is categorized into three levels, “High”, “Moderate”, and “Low”, depending on the intensity, duration, and number of days of physical activity performed per week [22]. Since the association between IPAQ-SV and physical activity by IPAQ-SV had been investigated in a previous study on psychological distress during the COVID-19 pandemic in university students [14], this index was also used in this study.

### 2.3. Statistical Analysis

A simple tabulation of each questionnaire item was performed. This study hypothesizes that the psychological distress of university freshmen who have lived through the COVID-19 pandemic is related to all current living conditions. To test this hypothesis, Pearson’s chi-squared test was used to determine the associations between psychological distress and living conditions, exercise habits, and physical health status of university freshmen during the COVID-19 pandemic.

Binomial logistic regression analysis (increasing-variables method) was conducted on significant items to extract the factors of psychological distress. The dependent variable was the presence or absence of psychological distress according to their total K6 score, with a cut-off value of 5 points. Explanatory variables were living conditions, such as lifestyle, exercise habits, and physical health status. Among the explanatory variables for the questionnaire items A–C and J–L in Table 2 were dummy variables with “Yes” = 0, “No” = 1. On the other hand, items D–F, H, I, M, and N were dummy variables with “No” = 0, “Yes” = 1. Gender was a dummy variable with “Male” = 0, “Female” = 1. Adjusted odds ratios and 95% confidence intervals (95% CI) for each explanatory variable were calculated. The significance level was set at *p* < 0.05 for all items. Statistical analysis software IBM SPSS version 28 was used for this analysis.

## 3. Results

### 3.1. Study Participants

Participant characteristics are shown in Table 1. Of the 2536 subjects included, 1317 (70.2%) were male and 524 (29.8%) were female, and the mean age was 18.1 (±0.4) years. According to K6 scores, 1841 persons (72.6%) had psychologically “no distress” and 695 (27.4%) were determined to “have distress”, of which 354 (14.0%) scored 5 to 8 points, 209 (8.2%) scored 9 to 12 points, and 132 (5.2%) scored 13 to 24 points. As shown in Table 2, faculty affiliation and residence style were not associated with psychological distress.

**Table 1:** The characteristics of study participants.

		*n*	(%)
Total		2536	(100.0)
Age (mean ± SD)	18.1 (±0.4)		
Gender			
	Male	1780	(70.2)
	Female	756	(29.8)
K6 Scores			
	0–4	1841	(72.6)
	5–8	354	(14.0)
	9–12	209	(8.2)
	>13	132	(5.2)
Faculty Affiliation			
	Engineering	832	(32.8)
	Business Administration and Information Science	284	(11.2)
	International Studies	118	(4.7)
	Humanities	373	(14.7)
	Bioscience and Biotechnology	384	(15.1)
	Life and Health Sciences	373	(14.7)
	Contemporary Education	172	(6.8)
Residence Style			
	Living with others	2157	(85.1)
	Living alone	379	(14.9)

**Table 2:** Relationship between psychological distress and basic attributes, lifestyle, exercise habits, and physical health status.

			Psychological Distress Classified by K6 Scores						*p*-Value
			No Distress		Have Distress				
			*n*	(%)	*n*	(%)	*n*	(%)	
			1841	(72.6)	695	(27.4)	2536	(100.0)	
	Gender	Male	1317	(74.0)	463	(26.0)	1780	(70.2)	0.016 *
		Female	524	(69.3)	232	(30.7)	756	(29.8)	
Faculty Affiliation									
	Engineering		626	(75.2)	206	(24.8)	832	(32.8)	0.056
	Business Administration and Information Science		201	(70.8)	83	(29.2)	284	(11.2)	
	International Studies		82	(69.5)	36	(30.5)	118	(4.7)	
	Humanities		257	(68.9)	116	(31.1)	373	(14.7)	
	Bioscience and Biotechnology		263	(68.5)	121	(31.5)	384	(15.1)	
	Life and Health Sciences		281	(75.3)	92	(24.7)	373	(14.7)	
	Contemporary Education		131	(76.2)	41	(23.8)	172	(6.8)	
Residence Style									
	Living with others		1574	(73.0)	583	(27.0)	2157	(85.1)	0.310
	Living alone		267	(70.4)	112	(29.6)	379	(14.9)	
Questionnaire item		Answer							
A	Member of an athletic club, or intention to join an athletic club	Yes	793	(76.4)	245	(23.6)	1038	(40.9)	<0.001 **
		No	1048	(70.0)	449	(30.0)	1497	(59.0)	
B	Intention to be physically active during university life	Yes	1387	(74.5)	475	(25.5)	1862	(73.4)	<0.001 **
		No	454	(67.4)	220	(32.6)	674	(26.6)	
C	Thinks exercise is good for physical health	Yes	1814	(73.0)	672	(27.0)	2486	(98.0)	0.003 **
		No	27	(54.0)	23	(46.0)	50	(2.0)	
D	Feels like not getting enough exercise	No	382	(77.3)	112	(22.7)	494	(19.5)	0.009 **
		Yes	1459	(71.4)	583	(28.6)	2042	(80.5)	
E	Feels sleep deprived	No	1658	(75.3)	545	(24.7)	2203	(86.9)	<0.001 **
		Yes	183	(55.0)	150	(45.0)	333	(13.1)	
F	Weight gain or loss of more than 3 kg in the past month	No	1660	(73.6)	594	(26.4)	2254	(88.9)	<0.001 **
		Yes	181	(64.2)	101	(35.8)	282	(11.1)	
G	Physical activity level according to IPAQ	Low	601	(71.5)	239	(28.5)	840	(33.1)	0.383
		Moderate	738	(72.1)	285	(27.9)	1023	(40.3)	
		High	502	(74.6)	171	(25.4)	673	(26.5)	
H	Feels communicates less with others at school or home than before the COVID-19 pandemic	No	1401	(75.2)	462	(24.8)	1863	(73.5)	<0.001 **
		Yes	440	(65.4)	233	(34.6)	673	(26.5)	
I	Feels more complications (conflict, etc.) in relationships at school and home than before the COVID-19 pandemic	No	1514	(75.6)	489	(24.4)	2003	(79.0)	<0.001 **
		Yes	327	(61.4)	206	(38.6)	533	(21.0)	
J	Finds it easy to talk to family members about worries and anxieties	Yes	1527	(77.1)	453	(22.9)	1980	(78.1)	<0.001 **
		No	314	(56.5)	242	(43.5)	556	(21.9)	
K	Finds it easy to talk to schoolteachers about worries and anxieties	Yes	961	(79.8)	244	(20.2)	1205	(47.5)	<0.001 **
		No	880	(66.1)	451	(33.9)	1331	(52.5)	
L	Finds it easy to talk to friends about worries and anxieties	Yes	1581	(76.3)	491	(23.7)	2072	(81.7)	<0.001 **
		No	260	(56.0)	204	(44.0)	464	(18.3)	
M	Currently experiencing certain physical symptoms	No	1429	(78.7)	387	(21.3)	1816	(71.6)	<0.001 **
		Yes	412	(57.2)	308	(42.8)	720	(28.4)	
N	Currently undergoing treatment for an illness	No	1771	(73.5)	638	(26.5)	2409	(95.0)	<0.001 **
		Yes	70	(55.1)	57	(44.9)	127	(5.0)	

**: *p* < 0.01; *: *p* < 0.05; COVID-19: novel coronavirus disease 2019.

### 3.2. Relationship between Psychological Distress and Living Conditions, etc.

The associations between psychological distress and attributes, living situation, exercise habits, physical health are shown in Table 2. The results showed that females were more likely to have psychological distress than males. The students who answered “yes” to the questionnaire items “I am a member of an athletic club or intend to join it”, “I am willing to be physically active in my university life”, and “I think that exercise helps my physical and mental health” had less distress as compared to those who answered “no” to these items (Table 2, A–C).

Students who felt they suffered from lack of exercise or lack of sleep, and those who had gained or lost more than 3 kg in the past month, were also more likely to have distress (Table 2, E and F). As for the level of physical activity using IPAQ-SV, 673 (26.5%) of the students answered “High”, 1023 (40.3%) answered “Moderate”, and 840 answered “Low” (33.1%); no significant differences were found between physical activity level and psychological distress (Table 2, G).

There was a high proportion of students who reported having distress in the form of complications (conflicts, etc.) in relationships at school or in their families, etc. and those who felt their degree of communication with others had reduced under the COVID-19 pandemic compared to pre-pandemic life (Table 2, H–L). Similarly, those who reported difficulty in discussing their worries and anxieties with their family, schoolteachers, and friends during the COVID-19 pandemic were also more likely to be distressed.

In terms of the relationship between psychological distress and physical health status, those who had some physical symptoms or an illness for which they were currently receiving treatment were more likely to have psychological distress (Table 2, M and N).

### 3.3. Living Conditions That Contribute to Psychological Distress

Table 3 shows the results of a binomial logistic regression analysis of the factors contributing to psychological distress among new college students.

The response “I am (or will be) a member of an athletic club” (odds ratio [OR], 1.327; confidence interval [CI], 1.093–1.611; *p* = 0.004) was a factor that prevented psychological distress. In addition, the following responses were also factors that averted psychological distress: “I feel comfortable talking to my family about my worries and anxieties” (OR, 1.721; CI, 1.340–2.211; *p* = 0.000), “ I feel comfortable talking to my schoolteachers about my worries and anxieties” (OR, 1.471; CI, 1.183–1.829; *p* = 0.000), and “I feel comfortable talking to my friends about my worries and anxieties” (OR, 1.588; CI, 1.225–2.059; *p* = 0.000). Conversely, the following responses were found to be linked to psychological distress: “I feel sleep-deprived” (OR, 1.935; CI, 1.498–2.500; *p* = 0.000), “I have gained or lost more than 3 kg in the past month” (OR, 1.379; CI, 1.039–1.830; *p* = 0.026), “Compared to before the COVID-19 pandemic, I feel that there is less communication at school or home” (OR, 1.292; CI, 1.018–1.640; *p* = 0.035), “I feel that I have more complicated (conflicting, etc.) relationships at school or home than before the COVID-19 pandemic” (OR, 1.815; CI, 1.408–2.340; *p* = 0.000), “I currently have some physical symptoms” (OR, 2.503; CI, 2.053–3.050; *p* = 0.000), and “I am currently being treated for a disease” (OR, 1.856; CI, 1.254–2.748; *p* = 0.002).

**Table 3:** Odds ratios of living conditions contributing to “psychological distress”.

	Odds Ratio	Confidence Interval		*p*-Value
		Lower Limit	Upper Limit	
Is or will be a member of an athletic club ^a^	1.327	1.093	1.611	0.004 **
Feels sleep deprived ^b^	1.935	1.498	2.500	<0.001 **
Weight gain or loss of more than 3 kg in the past month ^b^	1.379	1.039	1.830	0.026 *
Feels communicates less with others at school and home than before the COVID-19 pandemic ^b^	1.292	1.018	1.640	0.035 *
Feels more complications (conflict, etc.) in relationships at school and home than before the COVID-19 pandemic ^b^	1.815	1.408	2.340	<0.001 **
Finds it easy to talk to family members about concerns and worries ^a^	1.721	1.340	2.211	<0.001 **
Finds it easy to talk to schoolteachers about problems and concerns ^a^	1.471	1.183	1.829	<0.001 **
Finds it easy to talk to friends about problems and concerns ^a^	1.588	1.225	2.059	<0.001 **
Currently experiencing certain physical symptoms ^b^	2.503	2.053	3.050	<0.001 **
Currently undergoing treatment for an illness ^b^	1.856	1.254	2.748	0.002 **

**: *p* < 0.01; *: *p* < 0.05; ^a^: “Yes” coded 0, “No” coded 1; ^b^: “No” coded 0, “Yes” coded 1.

## 4. Discussion

This cross-sectional study investigated the degree of psychological distress and its predictors using the K6 scale in 2536 freshmen who entered university during the COVID-19 pandemic. Although psychological distress was not particularly high during the pandemic, motivation to exercise, pandemic-induced loneliness and relationship changes along with physical health issues influenced psychological distress.

There were 695 students (27.4%) with a K6 score ≥5, i.e., “with psychological distress.” This result is slightly lower than the 28.5% of students with a K6 score ≥5 in a survey conducted among Japanese medical students in March 2020 [4]. In surveys of Japanese 20- to 64-year-old in 2010 and 2007 before the COVID-19 pandemic, the percentage of those with a K6 score ≥5 was 28.7% and 28.9%, respectively, while in March 2020, after the outbreak of the pandemic, the proportion with a K6 score ≥5 was 51.5% [20]. Noteworthily, the proportion with a K6 score ≥5 was 48.1% in April–May 2020, when compulsory lockdowns were implemented in Japan [17]. In comparison to these numbers, the university freshmen in this study did not show a higher K6 ≥5 ratio, so it is unlikely that psychological distress was exacerbated. The university freshmen of the class of 2022 spent most of their high school years during the COVID-19 pandemic that began in 2020. Thus, it was expected that they would have experienced a greater psychological burden due to activity restrictions and online education. However, mid-April 2022, when this survey was conducted, marked the beginning of their new academic lives after overcoming the COVID-19 pandemic and successfully deciding on a career path (i.e., one that included higher education). In addition, it may be assumed that this batch of students had become accustomed to the COVID-19 pandemic, which had begun early 2020, and were better able to cope with changes in their lives. For these reasons, we surmise that psychological distress may not have worsened by much. A survey in the United States also reported that psychological distress decreased with the passage of time after the onset of the pandemic [23]. In addition, it was predicted that the risk of serious illness due to COVID-19 among healthy teenagers is low [24], and the knowledge of this fact had reduced fear of severe illness. Furthermore, the fact that the sixth wave of infection had subsided and that behavioral (activity, etc.) restrictions had been abolished may have prevented the worsening of psychological distress. Nevertheless, we cannot ignore the fact that 132 (5.2%) of the students with psychological distress had severe mental disorders (K6 ≥ 13) and it is necessary to implement some countermeasures for these students. It is possible that the surveyed group of students did not devote much time to physical activity just before entering university due to restrictions on club activities and preparation for entrance examinations.

Interestingly, we found that there was no relationship between the amount of daily physical exercise and the presence of psychological distress among university freshmen. By contrast, the results of the present study suggest that being in, or thinking about joining, an athletic club and/or the willingness to exercise regularly reduces psychological distress. It can be considered that psychological distress is related to the willingness to exercise and/or communication with others in an athletic club rather than the actual amount of physical activity itself. A survey of Polish teachers during the COVID-19 pandemic showed an association between psychological distress and physical inactivity and mild depression [25]. In a study of Chinese university students, daily exercise has been proved to be a factor in controlling their anxiety [26]. Other studies have reported that psychological health has been hampered by reduced physical activity during the COVID-19 pandemic [18,19,27,28]. In addition, physical activity during the COVID-19 pandemic affects the awakening quality [29], and aerobic exercise has been shown to improve immune and respiratory system functions, which is effective in combating COVID-19 [30]. Therefore, active exercise is recommended during the spread of COVID-19, and universities should consider increasing opportunities for exercise as well as encouraging and supporting athletic teams. Although the university surveyed offers a compulsory physical education practical-skills class for first-year students and a number of elective sports practical-skills courses, there is a further need to devise ways to improve physical activity on campus, such as establishing physical activity programs that can be easily participated in as recreational activities apart from the regular academic curriculum.

The students experiencing sleep deprivation are at nearly double the risk of greater psychological distress compared to those who are not. A March 2020 survey of Japanese population aged 20 to 64 years also noted an association between sleep deprivation and psychological distress [20]. There are numerous reports of an increase in sleep problems during the COVID-19 pandemic [31,32,33]. Thus, it is necessary to educate students about the importance of sleep and provide counseling services for students with sleep problems. It has also been reported that decreased physical activity during the COVID-19 pandemic also affected sleep quality [15]. Therefore, we believe that encouraging physical activity will lead to the resolution of sleep problems as well as improve psychological distress, directly or indirectly.

In the present study, weight gain or loss of more than 3 kg in the past month was associated with the presence of psychological distress. It was hypothesized that significant weight gain or loss could be an indicator of psychological health; both significant weight loss and weight gain could be signs of depression [34,35]. Since measuring and tracking weight is an easy-to-perform health observation, it should be watched closely.

The subjects in this study experienced less communication and a worsening of relationships during the COVID-19 pandemic compared with the pre-pandemic period. They also felt that it was difficult to discuss their anxieties and worries with those around them (family, friends, and schoolteachers), which may have increased their psychological distress. Behaviors to avoid the “3Cs” (closed spaces, crowded places, and close-contact settings), which are measures to prevent the spread of infection and include measures such as school closures and refraining from going out, also reduce social contact. Therefore, it can be observed that students would increasingly feel lonely due to social isolation. A survey of workers and pregnant women in Japan has indicated an association between loneliness and psychological distress during the COVID-19 pandemic [36,37]. During the compulsory lockdown in Japan, high loneliness and poor interpersonal relationships were associated with psychological distress [17]. According to a study among German university students, loneliness increased significantly after the first and second lockdown and was associated with anxiety and depressive symptoms [38]. A survey of Iranian medical students found a link between loneliness and psychological problems [39]. Although the present study was conducted in the absence of a lockdown or other restrictions, the results indicate that poor relationships and loneliness during the COVID-19 pandemic can affect psychological distress. While the university where the study was conducted has facilities for student counseling, it is still important to identify students with distress and loneliness and direct them to these services. It is also necessary to focus on resuming or making new plans for recreational and other events that would build interpersonal relationships among students and would support extracurricular activities.

Having any physical symptoms was associated with a 2.5-fold increased risk of psychological distress. The students that had a current illness and were undergoing treatment were at nearly double the risk of psychological distress. It was reported that migraines and fatigue were worsened by distress during the COVID-19 pandemic [40]. Patients with painful chronic illnesses or chronic pain are found to be at risk for psychological distress, depression and anxiety disorders [41,42,43]. This suggests that there is an association between physical health and the worsening of psychological health during the COVID-19 pandemic. We believe that confronting existing physical ailments along with improving pain and other physical symptoms will lead to an improved psychological well-being. Therefore, the university should support regular health checkups and proactive medical examinations and treatment.

## 5. Limitations

This study has the following limitations: (1) It is a survey of only one university which the researchers could investigate, and thus, does not provide a general or comprehensive assessment of psychological distress in students. (2) Careful attention should be paid to the the point that differences exist in the male-to-female ratio of participants in this study. (3) Attention should also be paid to the difference in the ratio of those who have and those who do not have psychological distress.

However, since the university where the survey was conducted is a general university with faculties in a wide range of fields that and enrolls students from all over the country, we believe that the sample could encompass differences in students’ academic orientation and regional characteristics.

## 6. Conclusions

In this study, psychological distress among college students during the COVID-19 pandemic was not significantly worsening, but there is a need to address the certain number of students who are in severe distress. It was found that unwillingness to join athletic clubs, lack of sleep, significant weight gain or loss, poor interpersonal relationships, loneliness, and physical discomfort were factors associated with psychological distress. Therefore, the university should make efforts to enhance physical activities and activities to stimulate interpersonal relationships, as well as support extracurricular activities to encourage students to manage their physical condition, including sleep and weight. In addition, it is necessary to take measures that can directly deal with psychological distress, such as conducting stress checks, creating a system for early detection of mild cases and connecting them to counseling, and establishing various consultation channels that are easily accessible to students. Students should be encouraged to actively utilize the mental and physical health counseling services that are easily accessible at the university.

## Data Availability

Not applicable.
